# Re-territorialising skills? Insights from ethnography on solidarity-economy food activism

**DOI:** 10.1007/s11625-024-01527-0

**Published:** 2024-07-12

**Authors:** Cristina Grasseni

**Affiliations:** Cultural Anthropology, Leiden University, Pieter de la Court Building, Wassenaarseweg 52, 2333 AK Leiden, The Netherlands

**Keywords:** Skills, Trust, Transparency, Solidarity economy, Proximity, Food activism

## Abstract

The article addresses the role of citizens in setting up short food chains in the framework of the solidarity economy movement in Lombardy, Italy. On the basis of ethnographic fieldwork with solidarity economy activists and longitudinal ethnography (2009–2023), the article critically analyses solidarity-driven experimentations with local food systems, including direct bulk-buying from farmers and setting up a Participatory Guarantee System (PGS) to self-certify organic agriculture quality standards as attempts at (re)territorialising the food chain. This implies innovative relationships and practices connecting farmers and consumers in a role for citizens as ‘co-producers’. Hurdles and dilemmas about trust, skill, and transparency question which role citizens can take as levers of change. Addressing one of the questions posed in this special issue (“what is the place of citizens and collectives in innovative supply chains?”), the article reflects on what kind of skills are required, and perhaps lacking, for a more active involvement of citizens in ‘co-production’. The article focusses on (1) how trust between producer and consumer is supposed to be engendered in relations of proximity, (2) the reciprocal performance of expected roles among consumers and farmers, (3) the difficulties of evidencing reciprocal transparency without negotiating mutual reskilling.

## Introduction

Building on longitudinal ethnography with solidarity economy networks in Lombardy (Italy), this article critically analyses “the place of citizens and collectives in innovative supply chains” to “(re)-territorialise agriculture” (Loodts et al. 2022, p. 1716). The social actors whose viewpoint and practices of “responsible consumption” (Forno et al. 2018) I portray are members of Italy’s Solidarity Purchase Groups (*Gruppi di Acquisto Solidale*, henceforth GAS). I address the relationship they wish to develop with food producers in their attempt to move “beyond alternative food networks” (Grasseni 2013). The key concepts covered are: (1) trust, in particular how Italian solidarity economy networks wish to ‘co-produce’ trust in their relationship with local producers; (2) skills, in particular how roles are performed and skills are displayed in reciprocal expectations between militant consumers and producers; (3) the problematic notion of ‘transparency’, in particular in the light of the food activists’ lack of “skilled vision” (Grasseni 2022). Examples will be taken from both ongoing and inactive projects, which the author has witnessed in their evolution since 2009, both firsthand and remotely.

## Literature review and analytical framework

Scholarship about alternative food networks is vast. I focus on grassroots, consumer-driven, collective projects (Piccoli et al. 2023; Vittersø et al. 2019; Cuéllar-Padilla et al. 2022). Chiffoleau et al. (2017), building on their research on open-air markets as “devices” favouring social relations that leverage new consumption practices, propose that zooming into social relations helps understand how (and if) short food-chains are transformative. Forno et al. (2016) similarly suggest that GAS function as “citizenship labs”, where members acquire skills for lifestyles change.

Scholarship on solidarity-driven short food-chains focusses on their closeness with social movements (Weiner and Forno 2020), on equitability (Zollet et al. 2021), or the role of food non-profits (DuPuis and Christian 2023), but less often on a cultural analysis of their discourse and practices. In the framework of an anthropological analysis of solidarity, Smith and Grasseni (2020) compared Istrian wine-makers’ networks of self-help with an experimental Lombard Participatory Guarantee System, concluding that informal economic relations entail reciprocal feelings of responsibility, but also social pressure. Discussing food justice in community supported agriculture’, Parot et al. (2023) distinguish between “charitable” and “emancipatory” “social support actions”, and connect trust with transparency. For example members of a community-supported agriculture scheme apply for solidarity-based sliding scales, as well as openly “bidding” (or “pledging”) for their monthly contribution towards the CSA running costs (Parot et al. 2023, pp. 7–8).

However, it is not often investigated what kind of reciprocal expectations solidarity-based economic relations engender, nor what are the relational implications of transparency being ostensibly ‘performed’. In the following review, I focus in particular on issues of trust, skill, and transparency.

While trust features in descriptions of “trust-based supply chain relationships in urban local food systems” (Nakandala et al. 2020, cf. also Lohest et al. 2019), it is rarely articulated what trust actually amounts to, beyond long-term, “direct relationships” and “shared moral value ascribed to the food” (Delibas 2021, p. 2). Investigations into how trust is built mention “openness” (Braun et al. 2023, p. 427) and “transparency”: for example a farmer re-contacting the consumers of a Peasant’s Box scheme having “found a spoiled batch, showing transparency and a willingness to ensure consumer satisfaction” (Delibas 2021, p. 11). Thorsøe and Kjeldsen (2016, p. 157) consider trust “a mechanism that creates coherency and facilitates co-operation in the food network”. Consistency within the organization, reasoned agreements, routine, and reflexivity make the mechanism work (Thorsøe and Kjeldsen 2016, pp. 166–168). Nakandala et al. (2020) investigate trust-based relationships from a retailer perspective. Despite being in relations of very uneven power, in retail-grower relationships “non opportunistic behaviour”, “perceptions of mutual justice”, and “fairness in ordering” (p. 876) are built over time both horizontally and vertically. Therefore, “collaborative”, “non-contractual” “supply chain relationships” would ensue from “the perceived fairness in social exchange” (p. 872) and from “mutual understanding of the advantages and constraints of local fresh-food systems” (p. 875), including the “high uncertainty in supply volumes and quality” (p. 877). Giampietri et al. (2018) find that “a sense of trust” is “built on a shared know-how and a mutual understanding with farmers” (p. 161). Being interested in predicting consumer purchase, they propose trust as a marketing strategy for short food-chains without a critical interrogation of what trust entails in terms of expected roles.

Transparency and trust are often associated: in scholarship on solidarity economy, Loh and Shear (2022) prefigure that new economic subjectivities are engendered in an “ontological politics” based on “relations of trust and openness” (Loh and Shear 2022, p. 1216). Looking at community supported agriculture in Germany, Zoll et al. (2023) contrast “opaque value chains” with “transparent production or social interaction between consumers and producers” that would “enable consumers to observe where their food is coming from”, concluding that “producers’ willingness to be transparent already signals trustworthiness to CSA members and is more important to members than formal signals” (p. 709). However, Koensler (2023) problematizes *how* transparency is expected, required or conjured in self-defining horizontal relationships. He opposes “emancipatory transparency-making” to transparency as a “new universal ideological formation”, a “new regime of governance” that actually disguises “unequal encounters”. Through ethnographic vignettes, he shows how contrasting understandings of “how transparency should be practiced” is unclear, debatable, and creates “friction” (cf. Tsing 2004).

Scholarship also rarely focus on which skills are needed to realize the promise of solidarity-driven and sustainable food systems vis-à-vis the many logistic, relational, and organizational challenges (but see Gillette and Grasseni 2022; Loodts et al. 2022; Grasseni et al. 2022). Contessi (2015a, b) for example argues that organic certification, whether institutional or participatory, does not sufficiently address the issue of soil contamination. Contessi and Grasseni (2019) insist on the lack of environmental and agronomic expertise in the Participatory Guarantee System that will feature also in this article. Thomas et al. (2020) highlight the “key roles of economic and social organization”, as “alternative knowledge and skills about food are shaping themselves” in “current practices along eaters’ trajectories and in social networks” (p. 27). They distil a research agenda which, amongst others, prioritises the need for “reconnection paradigms” (Lamine et al. 2019) across food science, plant and animal sciences, and the social sciences (Thomas et al. 2020, p. 37).

This article critically reconsiders trust, skill, and transparency in solidarity-economy food activism. Over three ethnographic sections, I will discuss (1) the ambiguities of ambitions to reterritorialise food-chains based on trust and cultural notions of proximity; (2) militant consumers’ (lack of) skills and expertise, and their expectations about their relationship with producers, which may lead to the reciprocal performance of expected roles; (3) the problematic notion of ‘transparency’ in the light of the food activists’ lack of the “skilled vision” needed to recognize environmental hazards and performative role-play.

The case study discussed regards solidarity economy networks in Lombardy, a northern Italian region with an agricultural tradition that dates back to the Middle Ages (Sereni 1977, pp. 259–270; Corti 2005). The Lombard plains boast Italy’s most intensive agricultural production, including dairy farming and cattle progeny selection. Established on an industrial scale in the American Midwest in the late nineteenth century, and propagated in Europe with added momentum after WWII, the drive for breed improvement and agricultural intensification has de facto marginalized smallholders and family farms, which in 1961 still counted 4.3 million agricultural enterprises in Italy, the majority of which registered as self-standing farmers (ISTAT 2023).

Solidarity Purchase Groups (GAS) advocate solidarity for “the environment, for food producers, and for each other” (Documento Base dei GAS 1999, pp. 6–7). Solidarity builds on the cultural sentiment of reciprocal care one feels for one’s neighbour. This symbolic and relational semantics of proximity complexifies the notion of “triple proximity” in food systems (defined in terms of distance, relations, and values: Eriksen 2013), promising a caring relationship, albeit not without ambivalences (Geschiere 2009). GAS group members convene periodically to self-organize (mainly food-) provisioning on a collective basis. They share information about available producers, collect orders for the entire group, place them and collect them. In the name of solidarity, GAS also collaborate with their providers, for example to organize farmers’ markets. Through these “relations of regard” (Offer 1997, 2007, pp. 75–99), this movement wishes to ‘co-produce’ food not by farming, but rather by enabling fairer deals with local farmers (Tavolo RES 2010).

Mapping the solidarity economy movement is difficult because there is no formal national registry and many initiatives spontaneously open and close. When they were first mapped in 2012, there were at least 450 Solidarity Purchase Groups (GAS) in Lombardy (a region of about 10 million inhabitants) involving about 7,000 families (Forno et al. 2016; Signori and Forno 2019). GAS self-inventory in the province of Bergamo (about one million residents), currently counts 49 groups coordinated in one network, ReteGasBergamo.^1^ A 2018 nationwide survey on “responsible consumption” (including Fair Trade and responsible tourism along GAS) found that 10.6% of about one thousand respondents purchased at least some of their daily products through GAS groups (Forno et al. 2018, p. 2).

Solidarity economy networks attempt to (re)territorialise agriculture by re-engineering supply chains, so that food can be procured in bulk from small-scale operators. The expectation is that this will increase informality over formalization, to the benefit of farmers rather than so-called middle men and large distribution chains (Grasseni 2013). This expectation synergizes with cultural and political preferences for local foods (Grasseni 2014). In some cases, GAS devise further participatory economic practices than just bulk-buying in groups. In this case, several GAS groups become building blocks in self-organized ‘Districts’ and ‘Networks’ of solidarity economy. A District of Solidarity Economy (DES) is a consumers-initiated collaboration with (a network of) producers, or with associations and institutions. While each GAS group consists of a small number of families, through a DES they can organize in ‘networks of networks’ in order to have sufficient lobbying or purchase power for dedicated projects, for example a short food chain (Biolghini 2009). A Participatory Guarantee System (PGS) and Bergamo’s District of Social and Solidarity Economy (DESS) are two projects that the Lombard GAS movement in the area I studied developed over time. In the “Conclusion and perspectives” section, I explain how they differ and how these projects (PGS and DESS) focus on different strategies for (re)territorialising food procurement in the Lombard solidarity economy movement.

Solidarity Purchase Groups and their Districts and Networks operate on important cultural and relational premises, which this article explores. The three sections following the methodology will each discuss one aspect of field observations on trust (1), skill (2), and transparency (3), in their sociocultural context. Section one introduces the notion of proximity in relation to trust; section two critically examines how farmers may perform expected roles and showcase traditional skills to convince food activists of their trustworthiness; section three critiques the idea of transparency in the light of consumers’ (lack of) “skilled vision” (Grasseni 2022). In these three field research sections, I use cultural analysis and ethnographic vignettes.

## Methodology

“Multitemporal research” is widely experimented with in anthropology (Howell and Talle 2012). In a longitudinal approach, ethnographers follow a social movement, or the development of a business sector, or a given social group, over several years and with a varied battery of methods. They can thus collect not only different, but changing perspectives over time, and can get a better understanding of the significance of a phenomenon in the context of an extended process or a much more complex system. Examples of methods in longitudinal ethnography include the ethnographic “revisit” (going back to a site of extensive fieldwork for a follow-up fieldwork), “diachronic research involving continuous and sustained engagement over time” (Berckmoes et al. 2021, p. 96) or multigenerational projects. In the case of this research, an initial period of participant observation as resident member of one Solidarity Purchase Group (GAS) in Bergamo, Lombardy, and as delegate in its province-wide network RETEGAS during 2009–2011 (Grasseni 2013) continued with follow-up visits in 2013–14, during which in-depth interviews were gathered with representatives of the solidarity economy movement in Lombardy. All the people I dealt with were made aware of my role as scholar. Being well aware that overlapping the roles of observer and participant is ethically complex, I kept myself at the margins of the organisation in 2009–2011. For example, I declined to candidate myself for coordinating roles to avoid contributing to shape the movement I wanted to describe (Grasseni 2013, pp. 166–167). As I left Italy in 2011, the possibility of such overlap ceased to exist. Upon my motivated request for research purposes, I was kept on the mailing list of the GAS network.^2^

Beginning 2012, doctoral research conducted under my supervision by Silvia Contessi compared various grassroots systems of food quality certification in Lombardy, including a then emerging Participatory Guarantee System project in the Lombard provinces of Como, Varese and Monza-Brianza (Contessi 2015a, b). Besides supervising the research and facilitating contacts with the solidarity economy network, I personally participated in one of the two field inspections attended by the Ph.D. candidate, together with the SPG committee, and in one of the four closed-doors meeting the Ph.D. candidate attended in the period 2013–2014. I continued to conduct in-person interviews and participant observation during follow-up visits to solidarity economy events myself, in the framework of the Wenner–Gren project *Seeds of Trust* (2013–14).

Since 2017, I resumed short-term visits to the solidarity economy networks of Bergamo in the framework of the comparative project *Food Citizens?* My sources of information for this period are my own observation during fieldwork visits, what participants told me in informal conversations and formal interviews, the analysis of public documentation, local and professional press, and formal and informal conversations with scholars and participants in the solidarity economy movement. Follow-up questions via email and zoom conversations complemented fieldwork evidence. Table 1 summarises the typology of sources consulted and their scale of action, as well as events attended over the period 2010–2023.

**Table 1:** Sources of ethnographic information

Period	Source	Scale of action
2009–2011	Active membership in a GAS group and GAS network	Bergamo province
2010–ongoing	Mailing list of solidarity purchase groups (GAS) network (with explicit permission)	49 groups in the Bergamo province
2012–2015	*Seeds of Trust* project and Ph.D. supervised on grassroots certification systems	Lombardy
2017–2022	Thematic analysis of magazines and newspapers on citizens’ participation in food sustainability	*L’Eco di Bergamo* and *Info Sostenibile* (Bergamo province); *Nuova Ecologia* (Italy)
2021–2023	Participation in public events organized by DESS BG (District of Social and Solidarity Economy of Bergamo)	Bergamo province
2017–2023	ERC *Food Citizens?* Project. Quarterly visits to Bergamo’s Farmers market ‘Market and Citizenship’. Four field visits to Turin	Bergamo, Turin
2019; 2022	Conferences of Bergamo Urban Food Policy Pact	Bergamo
2022–2023	In-depth interviews and conversations with 12 privileged interlocutors and follow-up correspondence with 5 privileged interlocutors	Scholars, activists, activist-scholars, and charismatic leaders in the solidarity economy movement in Piedmont and Lombardy

The method of longitudinal ethnography—namely an ethnographic observation of the same broad topic and networks of interlocutors over a long period of time—allowed ongoing conversations with stakeholders and research participants to develop over time. It brought into relief not only the importance of the relational context between researchers and informants, but also between food activists and producers.

### Trust in proximity

GAS members are “responsible consumers” (Forno et al. 2018) whose goal is to “co-produce” (Biolghini 2007) food and services for life necessities, in particular to enable fairer deals with local and small-scale food producers through bulk-buying directly from them. During my fieldwork, short-chain projects were being introduced also around solar energy, affordable dentistry, and telecommunication. Various ad hoc interventions supported food producers in post-disaster areas, for example after significant earthquakes in other regions of Italy, by placing sizable orders with producers in the affected area. An apt example of food short chain is *Spiga & Madia* (ear and trough)*,* ideated and managed by a network of GAS near Milan*.* This project connected a farmer, an organic mill and a network of bakeries to produce bread for 500 families with locally grown organic wheat. With a distance between the different nodes of the network barely exceeding 20 miles, this became a 0-mile short food chain model within the GAS movement (De Santis 2010).

Co-production is the language with which GAS activists designated these collaborations, prefiguring them as a form of ongoing relationship between producers and consumers. This long-term alliance may include the prospect of supporting conversions to organic farming, or the promise of setting up independent systems of quality certification. A participatory guarantee project was ideated within this framework by the Districts of Solidarity Economy (DES) of Como, Brianza and Varese in 2012. The international procedure for Participatory Guarantee System (PGS) has been codified by IFOAM (the International Federation of Organic Agriculture Movements, see May 2008) since 2004, and has been implemented for example in the *Ecovida Agroecology Network* of Southern Brazil (Rover et al. 2017). The DES project claimed to be the first PGS project in Italy and was set up with a seed grant from a regional bank foundation (CARIPLO), which funds social development projects.^3^

Its basic working mechanism depended on a guarantee committee which, according to the guidelines of the project, would receive and assess field data from an inspection group at regular intervals, after an initial visit sealing a memorandum of agreement. Both inspection group and guarantee committee would consist of one producer, one consumer and one expert. Crucially then, it would not be the case that a producer certifies oneself or is exclusively peer-certified, but rather that a peer-producer together with a consumer and a technical expert (an agronomist) would monitor farmers selling to the GAS network participating in the project. It was envisaged that this format should nourish ongoing conversations among diverse stakeholders (producers and consumers). Independent expertise would not be provided by a third-party company. For example, the inspecting committee I accompanied on their field visit in 2013 enrolled no independent technician but included a GAS member who was also an agronomist by profession.

Once I was admitted to taking part in the field visit as auditor, I received all the relevant documentation of the farm involved, including the production protocol and the signed declaration of intent. These artefacts are worth examining: importantly, the declaration of intent is not a contract binding the producers to the consumers. The cooperative farm in question was entering little more than a verbal agreement to follow a production protocol. Vice versa the network of GAS groups would only be morally bound to buying the crops. The objective of this informality was not only to avoid the costs of a certifying third party, but also to underline the reciprocal moral obligation of producer and consumers, beyond a formal transaction. It is not the *Guardia di Finanza*—the tax and customs police—nor the Ministry for Agriculture who would intervene to sanction any misconduct—as it would be, for example, in case of the mis-use of a protected denomination of origin label (PDO) or an institutional certification for quality assurance. Participatory guarantee was seen by solidarity economy activists as a means of making producer-consumers transactions more trustworthy by drawing legible, personal and meaningful connections between the food they buy and its makers.

The PGS network subsequently expanded in Lombardy with a further project in 2015 and 2016 under a new name, *C’è Campo* (there is field).^4^ When asked about the state of the art in 2023, five privileged stakeholders in the Lombard solidarity economy movement said that “the website is still online but the project is dormant”; “there has not been any progress on that front”; “in theory RIES should have taken it over from Tavolo RES”^5^ and “we still hope to reactivate it”. Some mentioned shortsightedness and occasional character incompatibility resulting in frictions within and across networks. The project was one of the few initiated in Italy and has been studied in recent scholarship as significant because of its consumer-driven setup (Piccoli et al. 2023). In the nationwide inventory of the Italian PGS schemes of Tuscany, Lombardy, Apulia, Campania and the cities of Rome, Parma and Bologna (Vittori 2018), the Lombard PGS scheme stood out for being ideated and driven by GAS consumers rather than by farmers. The fact that it was de facto suspended is instructive. For example, costs for paying agronomists in field visits were mentioned in 2023 as one of the deciding factors that slowed down the reprise of the project.

Confronting hard economic facts also sealed the destiny of other renowned DES projects, such as *Spiga & Madia* mentioned above. Their wheat fields were expropriated and dug over just the year preceding the 2015 Milan EXPO (themed ironically *Feeding the Planet*), as part of the plan to build an extension of Milan’s ring-road. The latter was fast-tracked as an urgent agenda item because of the logistic needs of the upcoming universal exhibition. As a result, *Spiga and Madia* had to rehouse their project in newly rented fields. A representative of *Spiga &Madia*, in August 2013, commented on eviction as a practically inevitable fact, in an area where land cost (then) 100,000 Euros per half acre: cultivating land in the Milanese plains is precarious, due to very short-term leases and high rents, he explained. The project nevertheless inspired other ongoing GAS-driven initiatives, for example *Piccola Filiera di Montagna della Farina e del Pane* (short bread-and-flour mountain food chain) in the Bergamo province. This is an organic short chain aimed at the agroecological cultivation of wheat in high lands, using a local organic mill and selling flour within the GAS network for self-baking, or bread baked in an organic oven. According to a public meeting I attended in April 2022, despite a distinction bestowed by the environmentalist association *Legambiente* (Legambiente 2020, p. 21), the hurdles remain the lack of affordable land to cultivate organically, the unpredictability of yields, the labour-intensity of small-scale operations, aggravated by mountain logistics, and the lack of (human and financial) resources. As commented by the project coordinator to the four other speakers, eight audience in person, and four audience online, “we are always the same few usual suspects”.

Reflecting on these experiences, it appears that among the challenges faced by the attempts of the Lombard solidarity economy networks to reterritorialise agriculture is that GAS activism requires a high degree of motivation and participation, and not only by militant consumers. GAS groups expect reciprocal engagement from their trusted producers. Even among a niche of dedicated activists, reasons for disenchantment were not lacking. The same interlocutor commenting on *Spiga &Madia* in 2013 praised DES Como, which initiated the PGS project, for being “capable of building real pieces of economy” according to solidarity principles, including setting up a workers’ cooperative with half a dozen fully employed staff. Nevertheless, my interlocutor remained sceptical of the role played by producers. Allegedly some of them relied on solidarity buyers as “some guise of social assistance”, but kept their standards low, instead of raising them as expected in GAS prefiguration. “The thing is, do our producers buy their food through a GAS? Are they themselves members of a GAS? Or do they go to the supermarket? They go to the supermarket, I tell you”—was the disgruntled conclusion*.* What we learn from this anecdote is that, in the eyes of this DES activist, this recreates the vicious circle of the so-called free market: subsidized large-scale farms and large distribution chains race prices to the bottom and undercut smallholders. Therefore, when small-scale farmers shop in supermarkets, they drive themselves out of business as they shop.

His reflection shows also how GAS activists wish not only to enable local farmers to stay afloat in a globalising market, but also aspire to *convert* them (to sustainable farming and to solidarity economy). This suggestive expression, often used in GAS language, might be both translated as a mere switch to organic (a technical procedure) but also as a fully-fledged *conversion* to the morals of solidarity economy. In order to discuss its anthropological significance, I connect it with the ideal of proximity. As I observed in GAS practice and discourse, solidarity often translates in the expectation of a producer who is literally a neighbour, namely someone living in one’s vicinity. Smallholders are named *produttori di prossimità* namely “producers nearby”. While what is straightforwardly meant is cutting down on food miles, what is expected in discourse and in the language of the movement’s documentation is much more than that: proximity is invoked in its full semantic spectrum of being one’s neighbour. In Italian, the word *prossimo* means neighbour (as in Christian teachings). *Prossimità* thus evokes being close, available, and open to entering a reciprocal relation. Another semantic sphere that is enrolled in *prossimità* is the proximity of kin, the idea of belonging, and rootedness. In sum, solidarity food activists expect to nourish a social relation over an extended temporal scale. While it is accepted that organic conversion is a learning process, it is also expected that this process is a form of conversion to the moral expectations of their partnering consumers. These expectations, however, do not necessarily match the perceived needs of the producers whom GAS activists wish to ‘convert’. In an interview conducted in 2023, two representatives of Bergamo’s District of Social and Solidarity Economy reflected that the PGS project became dormant because, with hindsight, it was perceived as redundant by the producers. Organic small-scale producers, it was explained, do not have difficulties placing their produce, for example through the orders of GAS groups, or online orders, or at farmers’ markets, because demand far outsizes offer. The time-intensive commitment to the PGS was therefore shunned because unnecessary.

Engineering a certification system meets both practical and relational hurdles (see also Koensler 2023 on the grassroots certification *Genuino Clandestino*). The Lombard PGS project started slowly and laboriously—and stayed small, despite its ambition. It initially enrolled 16 farms in 2011 (Contessi 2015a, b) and 15 in 2016 (Salvi and Vittori 2017). According to the project final report (2016), 17 field visits were filed, and 21 potentially interested new producers were mapped. In sum, when GAS activists set up solidarity economy projects, they require substantive engagement not only timewise, but also in relational and moral terms—not only from themselves but also from ‘their’ producers. Hence, the moral outrage of the GAS activists, complaining that producers don’t do their shopping themselves through a GAS, but go to the supermarket instead. Doing so, they are perceived by engaged consumers as betraying the moral alliance expected of “co-producers”. Producers, was noted by my interlocutor, “act” as marginal actors, performing the underdog as small-scale, local producers, only to enjoy “consumer’s choice” in a supermarket. This aspect is the focus of the following section.

### Performing roles

What we learn from the language of co-production is that involvement in a solidarity economy network prefigures a transformative process, meant not just as consumers’ reskilling (learning to cook, or homesteading, for example), but as ‘co-production’ of all the knowledge necessary to set up and manage a short food-chain from seeds to table. This involves acquiring new expertise for all those involved—producers and consumers—through a laborious learning process. In a context of demographic pressure comparable to that of the Netherlands, coupled with increasing land prices and soil pollution, Lombard food activists share an imaginary and a condition of deprivation (of skills, of information, or of meaningful access to it). Their discourses and practices of solidarity respond to this lived experience. For example, at the annual national meeting of GAS and DES in Osnago in June 2010, in a closed-doors working group entitled “From food safety to food sovereignty”, I raised the issue of the lack of expertise on food production and organic farming that I perceived about myself and my fellow GAS members. We were a self-summoned group of people, interested in sharing knowledge about one particular topic (as “experts” or as “knowledge seekers”). However, the “knowledge seekers” far exceeded the “experts”. In other words, far more participants were in search of skills than the ones having them (or presuming to have them).The GAS leader who chaired the session announced that a “knowledge bank” would soon be created to facilitate GAS reskilling*.* For such a knowledge bank, relevant know-how would not be understood as the cognitive or intellectual property of an individual, but of a collective. Consistently, most of the preparatory studies and documentation, which cost GAS and DES volunteers time and resources, were published online open access [see for example, De Santis (2006) on the project *Spiga & Madia*].^6^

In my experience, the initiators of these projects are not agronomists nor farmers, but rather IT experts, university researchers, political activists, teachers, office workers. They educate themselves and each other in their spare time about organic farming, ethical banking, critical consumption, non-violent leadership, as well as trade treaties and global inequalities.^7^ The documents published in preparation of the Lombard PGS project were well-informed and meticulously curated, based on relevant information (manuals, case studies, international literature, web sites, activist scholarship). The underlying tenet was that if expertise is transparently shared, solidarity-based economies and horizontal decision-making can ensue, harmonising the interests of producers (selling) and of consumers (buying safely and conveniently). The two iterations of the project wished to make the skills of farmers and of critical consumers converge in a transparent way.

While I further elaborate on ‘transparency’ in the following section, here I reflect on how the steps laid out were meant to accompany and facilitate this convergence, helping a producer progressing from candidacy to membership of the network, to playing the part of peer-reviewer for other producers. As evoked in the project title, for a Pedagogy of the Land (*Per una pedagogia della terra*), Freire (2000)’s liberation pedagogy and its radical practices of reskilling as a tool for social liberation belong among others to GAS cultural references. Solidarity economy activists also read scholars of the Degrowth or voluntary simplicity movements. Some have a Christian missionary background, some come from the radical left, and some combine both influences in original ways (Grasseni 2013). Serge Latouche and Euclides Mance for example—respectively, a French leader of the Degrowth movement and a Brazilian philosopher of the “network revolution”—have been guests of GAS groups for book presentations and public seminars.

Through critical pedagogy, solidarity economy activists wish to prefigure and facilitate transformative relations of co-production between consumers and producers, rather than mere transactions. This approach includes an understanding of their skills in the framework of situated learning (De Vita and Vittori 2022). “Learning as a process” features among the keywords of the PGS manifesto, implying that one learns with others, through participating in routines which include relational, logistic, and material features. Learning is then understood as a practical activity (Lave 1988), a progressive assumption of responsibility in roles that bring a novice from the margins to the core of a skilful activity (Lave and Wenger 1991).

I toured farms as a GAS member in 2010/2011, as part of producers-scouting activities. Even without a PGS scheme in fact, GAS members are routinely in charge of contacting and selecting farms, cooperatives or food manufacturers. In my experience, there were moments of orchestrated *exhibition* of proximity, a concept discussed above, and of skill. For example, during on-site farm visits, dialect might be used. Photographic or historical documentation about the farm was volunteered by farm owners. From these, we would be cued that the farm in question is historically rooted and authentically local. Some farmers exhibited the small scale of their operations with eloquent gestures. As in market transactions (cf. Bachis et al. 2016), open arms or pointing hands would wave at the cultivated fields or ration for bred animals. With the gestural and linguistic exhibition of simplicity, the producers we were visiting claimed both transparency and quality: “*tutta roba genuina*” (all genuine stuff). But one breeder, for example, was unable to tell us what the cow fodder was made of. Grains? Soy flour? GM or not? She couldn’t tell. Another producer, a horticulturalist working also for Slow Food, showed us the block of turf and seedling he bought, “all ready to go” into his terraced garden – but whether the turf would contain fertilizers and herbicide, he could not say.

The ostentation of traditional skills included showing private cellars for maturing *salami* and cured meat for themselves, to evidence that homesteading was a rooted and continuous on-farm activity. However, there were also misunderstandings and embarrassment in the reciprocal re-positioning of producers and consumers, moving from a formal transaction into a relationship of proximity. This was sometimes interpreted as openness to complicity. A farmer proposed to sell their crop to GAS for cash, as a way of avoiding tax. An agro-tourism restaurant attempted not to issue a receipt for a dinner the GAS held there. Presumably attempting not to report the transaction for VAT, they did not know that GAS activists consider (fiscal) transparency as an important standard. What we learn as readers from these anecdotes is that positing that the relationship of producer and consumer should be one of essential alliance rather than of natural competition is only the beginning step of a long and potentially fraught process of inter-locution. Solidarity economy activists expect to co-produce trust. However, the effect of bypassing the authority of auditing third-parties may well be that reciprocal expectations don’t match. The producers who were overly keen to evidence ‘genuine’ food production were speaking the language of authenticity and of tradition (cf. Crossland-Marr and Krause 2023), while not quite prepared for pointed questions about provenance of animal fodder. The producers who expected to find sympathetic complicity in their direct partners (perhaps seeing the state as a third party that should be bypassed, too…) found out that they were dealing with zealous tax-payers.

Farmers may find it difficult to be transparent to ethical consumers who have best intentions but little knowledge of their trade. The objective of expert practice is not only the production of objects (in this case crops) of a certain standard, but also the reproduction of the people who can appreciate such standards (Herzfeld 2004). In the case of the direct relationship that GAS activists wish to establish with producers, based on proximity, it remained unclear to which extent producers and consumers shared an understanding of which standards would be appreciated. In the next section I further discuss the limits of the ideology of transparency (Koensler 2023), in the light of my “skilled visions” approach (Grasseni 2022).

### Skilled visions

We have seen that, in PGS certification, delegation to auditors is replaced by direct evidence. However, evidence can only be appreciated by skilled experts who know what to look for. Vice versa, as noted by Venkatesan (2010) about craft weavers in rural India, performing skill is a role for a cultural broker, who needs to act appropriately within an “economy of recognition” (Cant 2018). In this case, we have seen how placing trust in proximity raised issues of reciprocal positioning, and to the (over)performing of expected roles. In this section we focus on the issue of transparency, vis-à-vis the need of prior skills in order to see evidence.

Since it is impossible to acquire skill without practice, mechanisms of social control of knowledge, such as guilds and academies, historically aim at controlling standards of acceptable practice and training. Craftsmanship and professions have clear apprenticeship paths such as the medical schools, where the transmission of skill goes hand in hand with the establishment of (formal and informal) authority. Competence thus encompasses not only information, but also relationships of authority – not all of which are meant to be transparent. Learning to skillfully navigate expert practice can be an ambivalent process: institutions can actually discourage learning as much as they enable it. Michael Herzfeld, in his ethnographic work on apprenticeship, noted that in master-apprentice relations, quick learning is not always appreciated. In some cases the novice learns to buy time and not to look too keen to learn, in case one’s master suspects the apprentice of stealing the trade. Vice versa, some masters will make learning a hurdle, as things too easily transmitted are not taken to heart: the master will hide the core business and the apprentice will “steal the business with one’s eyes” (Herzfeld 2004, p. 115). None of this is accommodated in the ideal scenario of co-production, where knowledge and trust would generate each other spontaneously, as consumers’ prefiguration meets farmers’ skills.

The notion of skilled vision has been successfully employed as an analytical tool to describe the prefigurative knowledge of agroecological farmers (Loodts 2023). Here, I use it to relativize the idea of transparency. Skilled vision could be described as the understanding of salient information about one’s practice, or the capacity to make what is visible *relevant.*Learning how to look at fields, crops, fodder, stables, and animals is a complex form of apprenticeship (Grasseni 2022). Even though GAS use on-site visits as a common tool to get to know one’s proximal producer and thus establish trust, consumers might lack the skills to see the evidence they are looking for—as in this last ethnographic vignette.

When we visited a PGS-candidate farm in July 2013, the PGS inspecting team, a PhD student and myself appreciated how forthcoming the farmer was. He entertained us for more than four hours, keen to ask for advice, being new to farming himself. We spent the first good hour listening to his narrative sheltering from a sudden downpour under a tin roof. The peer-farmer and the peer-consumer of the inspecting committee attended to his explanations. The two women took great care responding and sharing advice from their own experience, as an organic farmer and an agronomy technician, respectively. The fresh pile of composted soil was uncovered and handled, appreciating its softness and richness – a sure sign that the worms had done their job well and had been fed and sheltered appropriately.

However, the farmer was also candid about the fact that they had to do a lot of “cleaning up” because the land-owner, a hunter, had interred animal carcasses, dispersed empty cartridges in the nearby woods, and allowed the abandoned lot to become a dumping site, mainly of building materials. With the iron rods recuperated from the fields they could build an entire row of supports for beans. The doctoral candidate—an experienced environmental technician—was aghast imagining the potential soil pollution that could have ensued, and doubted the seriousness of the entire enterprise when no soil-testing was required after these conversations. Moreover, despite the fact that the farm was situated in an idyllic location at the feet of the mountains, according to her not enough attention was paid by the visiting party (and me) to the prominent chimney of a nearby factory, or to the closeness of a trafficked road. To her, we were hopelessly *blind* to damning evidence. While we praised the farmer for the initiative of recuperating materials, she saw the potential dangers of allowing toxic pollutants to enter the food chain.

Having conducted the field visit together, and having both participated in a subsequent meeting of the guarantee committee of the PGS project, she could explain to me in the light of her environmental expertise as an inspector for the public administration, that much more than meets the eye is to be found in (and especially under) agricultural land. For example, in her job she had personally uncovered acid muds, residues of oil refinement, and solid urban waste in maize fields that go to feed dairy cows. Even certified organic farming is regularly conducted with almost certainly polluted waters – she explained—such pollution is simply not measured (Contessi 2015a, b; Contessi and Grasseni 2019). The number of pollutants and their capacity for recombination is such that standard laboratories cannot detect them unless they are specifically looking for some pollutants in particular. Then, there is the decision of the operator. She offered the example of the spectrometer obtained from sampled water: does it mimic the curve of a model pollutant, or another, or none? Such comparisons are made *at sight*, she complained, as if this undermined objectivity. I offered to consider that when substituting this “subjective” decision of the operator with a standard instrument, the subjectivity of judgment is simply delegated to the act of calibration itself. Subjectivity is simply black-boxed– and thus becomes invisible. We discussed what objectivity means, a civil servant with the pressing need for working standards and myself—a social scientist used to the paradigm of the social construction of scientific knowledge. But neither of us felt satisfied nor safer: independently certified or not, ‘having to know what to look for’ did not add up with the impression that the controls were not as comprehensive as she would have recommended.

What we learn from this anecdote is that to a professional inspector, grassroots committees lacked the skilled vision to play the inspecting role. This was confirmed in her eyes by the conclusions they drew. Following our on-farm visit of July 2012, the Guarantee committee concluded in January 2013 that “the choice of developing a new agricultural activity deserves support, especially given its social contents. We share the double-sided opinion of the visiting committee: a sincere appreciation and the invitation to not act as *dilettanti*”.^8^ By using the word *dilettante* the committee criticizes the farmer, implying his practice was still below professional expertise. However the PGS certification was conceded, without a period of organic “conversion”, since the land had not been previously cultivated. Exploring the full semantic spectrum of “conversion” as suggested in “Introduction”, we see that the discourse of co-production prefigures conversion to organic farming as an ongoing salvific process. Practices are flawed, but a continuous learning process would eventually redeem the initial inexperience.

## Conclusion and perspectives

Investigating the discourse and practice of ‘co-production’ in Lombardy’s solidarity economy networks, I have highlighted how grassroots efforts to cultivate trust, skill and transparency encountered relational problems originating in the discrepancy between consumers’ and producers’ expectations. Consumers-driven GAS and PGS rely on expectations of ‘conversion’. Over “Introduction”, “Literature review and analytical framework” and “Methodology”, I have focussed on the role of cultural notions of proximity in GAS strategies of substituting direct relationship to third-party certification (1), which skills are showcased in the producers’ performance of expected roles (2), and which skills militant consumers need or lack (3). The consumers prefigurations of *converting* producers, rather than helping *articulate* and *negotiate* the expectations and commitments of *both* parties, projected unequal roles onto the producers, while on the other hand the latter did not know exactly how to interpret consumers’ ‘trust’. Their reciprocal expectations did not harmonize spontaneously. Consistent with what Koensler (2023) maintains, ‘clarity’ and ‘transparency’ can serve as idioms of hegemonic regimes even with best pedagogic intentions. The relational symmetry evoked in ‘co-production’ did not find a translation in field-practice and eventually was not attractive for producers, who did not massively subscribe to the PGS.

Beyond this, longitudinal ethnography allows to observe how the solidarity economy movement is adapting their discourse and practice over time. For example, the COVID-19 pandemic (2020–2022) led to the widespread acquisition of online tools to manage orders and deliveries. Among these, the online platform *L’Alveare che dice sì!* (http://www.alvearechedicesi.it) claims to facilitate direct transactions between local producers and ethical consumers but accepts that organizing this logistics must be a remunerated job, and levy costs for the service. An internal survey conducted by the District of Organic and Social Agriculture in the province of Bergamo (BioDistretto Bergamo https://www.biodistrettobg.it/) by the end of 2020 found that 76 out of 93 organic producers would be interested in participating in an online platform with logistics for home delivery.^9^ This revolutionises over 30 years of GAS practice based on face-to-face informal self-organisation. However, notions of proximity and trust remains key also to digital alternative food networks. Viciunaite for example, in a study of 768 consumers using a digital farmers’ market on Facebook in Norway, finds that ‘the most important user network attribute’ ‘was that people one trusts also shop’ on the same platform (Viciunaite 2023, p. 7).

Another significant evolution for the province of Bergamo is a newly founded District of Social and Solidarity Economy, which since September 2021 organizes cultural events, political debates and educational campaigns, for example on reducing food waste. Partnering associations include the environmentalist association Legambiente. DESS-BG also organizes the fortnightly farmers’ markets *Mercato agricolo e non solo*, namely open-air markets with added cultural events or meetings with local producers.^10^ The markets resulted from political and cultural convergence among the city’s many environmentalist, and cooperative associations, initiated in 2007 by Francesca Forno with the informal network Sustainable Citizenship.^11^

Finally, in the aftermath of Milan’s 2015 Expo and Urban Food Policy Pact, a Bergamo Urban Food Policy Pact was launched with input from the municipality and the university amongst others. The conference Agriculture and Right to Food (2019) presented a grassroots proposal for a regional law on social and solidarity economy in Lombardy (which however was not adopted by the Regional Council), and showcased Bergamo Green, a 2017 digital map of sustainable producers and consumer’s hubs in the province.^12^ With the following conference editions, the Bergamo solidarity economy movement found an institutionalized echo, and aligned with societal partners (such as Slow Food, the cooperative movement, and the organic agriculture movement) on a broader, less radical agenda of re-territorialising food procurement mainly through cultural, political and educational moral suasion, rather than by single-handedly engineering “real pieces of economy” (as a DES activist called their short food chain projects about a decade before).

Compared with the difficulties of GAS-driven short food chains, the Bergamo District of Social and Solidarity Economy chooses to focus on the skills and capacities already at hand among the social milieu of solidarity economy activists, namely education, organization, lobbying and networking, having learned that single-handedly setting up short food chains is time-consuming and requires different relational and technical skills. More field expertise, and less onerous forms of organization would be needed to move grassroots short chains “beyond the fringe” (cf. Utting 2015). As Laamanen et al. (2022) find, “scaling through institutions as prefigurative politics” pays in terms of permeating society with a cultural message, but does not necessarily bring about structural change in terms of setting up alternative (food) systems. At the same time as solidarity economy is becoming well known, privileged interlocutors in the movement admit to a sense of stagnation among its mostly middle-aged, middle-class members. This is well summarized by Cornaggia (2022, pp. 187–88): “many of the values they promoted have permeated culture, while the attempts to bring about a structural economic change in the relations of production and consumption, have had little impact”.

In sum, ethnographic insight into the expectations of trust, reskilling and transparency within solidarity economy networks that aim at (re)-territorialising agriculture through solidarity-economy driven short food chains shows that these ideals are generative and transformative from a relational and cultural point of view, but can be unsatisfactory in terms of economic change and practical efficacy. The solidarity economy movement has been successful in ushering a broader sensibility for sustainable, just and fair food among responsible consumers, but has not yet managed to change the mainstream modes of food procurement, nor to “create real pieces” of alternative economy. This is to be kept in mind when strategizing to “re-territorialise agriculture” (Loodts et al. 2022).

## Footnotes

1 http://retegasbergamo.it/. Last accessed 4th December 2023.

2 Latest personal communication with rete@retegasbergamo.it dating June 5th 2021.

3 http://www.lisolachece.org/progetto/garanzia-partecipata. Last accessed 4th December 2023.

4 https://www.lisolachece.org/progetto/sistema-partecipativo-di-garanzia-regionale/ and http://www.cecampo.org/ Last accessed 4th December 2023.

5 Rete Italiana di Economia Solidale (RIES) in 2020 took over from the national network of solidarity economy, Tavolo RES (https://economiasolidale.net/chi/tavolo-res. Last accessed 13th December 2023).

6 https://www.spigaemadia.it/il-progetto/ Last accessed 5th December 2023.

7 A break-down of professional profiles of GAS members and leaders is provided in the survey of GAS in the province of Bergamo by Forno et. al. (2013, 38–39).

8 Translated from the minutes (in Italian) of the PGS Guarantee Committee, 18th January 2013.

9 Personal communication, Cinzia Terruzzi, 26th November 2023.

10 The markets are offered on a fortnightly basis in Bergamo and Val Seriana (Albino and Alzano) and monthly in Val Imagna (Almenno).

11 CS’s spin-off association Market and Citizenship established the first markets.

12 http://www.bergamogreen.net/, a project by Francesca Forno. Last accessed 4th December 2023.
